# Role of IL-17 and Th17 Cells in Liver Diseases

**DOI:** 10.1155/2011/345803

**Published:** 2010-12-15

**Authors:** Linda Hammerich, Felix Heymann, Frank Tacke

**Affiliations:** Department of Medicine III, RWTH-University Hospital Aachen, Pauwelsstraße 30, 52074 Aachen, Germany

## Abstract

Unbalanced Th1/Th2 T-cell responses in the liver are a characteristic of hepatic inflammation and subsequent liver fibrosis. The recently discovered Th17 cells, a subtype of CD4^+^ T-helper cells mainly producing IL-17 and IL-22, have initially been linked to host defense against infections and to autoimmunity. Their preferred differentiation upon TGF*β* and IL-6, two cytokines abundantly present in injured liver, makes a contribution of Th17 cells to hepatic inflammation very likely. Indeed, initial studies in humans revealed activated Th17 cells and Th17-related cytokines in various liver diseases. However, functional experiments in mouse models are not fully conclusive at present, and the pathogenic contribution of Th17 cells to liver inflammation might vary upon the disease etiology, for example, between infectious and autoimmune disorders. Understanding the chemokines and chemokine receptors promoting hepatic Th17 cell recruitment (possibly CCR6 or CCR4) might reveal new therapeutic targets interfering with Th17 migration or differentiation in liver disease.

## 1. Introduction

In homeostasis, the liver is not only exerting various metabolic functions, but also serves as a central “immunological” organ. Blood coming from the gastrointestinal tract via the portal vein is rich of potential antigens derived from the gut-resident commensal microflora, ingested food, or also pathogens under infectious conditions. Immune cells that reside in or travel through the liver have the potential to initiate either (a) innate and adaptive immune responses in case of infections, for example, in response to lipopolysaccharide (LPS) or bacterial superantigens or (b) immunological tolerance to the vast majority of harmless antigens during homeostasis [1]. Following liver injury, induced, for example, by hepatitis viruses, alcohol abuse, or nonalcoholic steatohepatitis, inflammation is a pathological hallmark feature of chronic liver diseases. Sustained inflammation then promotes liver fibrosis and—as an end stage—liver cirrhosis or hepatocellular carcinoma [2].

Inflammatory responses upon liver injury comprise resident as well as infiltrating immune cells. It is well known that innate immune cells are important triggers of hepatic inflammation, because the liver is selectively enriched in macrophages (Kupffer cells), natural killer (NK), and natural killer T (NKT) cells [1]. In addition, the infiltration of monocytes upon liver injury is an important cellular mechanism to perpetuate chronic inflammation and to activate profibrogenic hepatic stellate cells (HSC) in mice and men [3, 4]. However, during conditions of chronic liver damage, adaptive immune cells are also crucially involved in the pathogenesis of hepatic inflammation. For instance, CD8^+^ and CD4^+^ T cells play important roles in hepatocellular damage, antiviral defenses (to hepatitis viruses), or autoimmunity [5, 6]. This paper will present the concept of different CD4^+^ T-helper cell subsets and summarize their proposed functions during liver diseases, with a focus on the current knowledge about the role of Th17 cells and their associated cytokines in liver inflammation in mice and men.

## 2. T-Helper Cell Subsets

CD4^+^ T-helper cells are major players in adaptive immunity. They provide help for antigen-presenting cells and CD8^+^ cytotoxic T lymphocytes to initiate and promote adaptive immune responses. Activation of CD4^+^ T cells is critical for the elimination of many invading pathogens, but inadvertently they can also become responsive to self antigens, thus leading to autoimmune diseases. In order to prevent this, the differentiation and activation of CD4 T-helper cells has to be tightly regulated.

**Figure 1:**
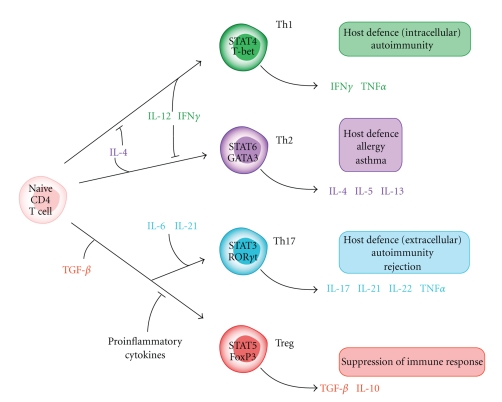
*Differentiation of CD4^+^ T-cell subsets (in mice). *Upon activation, naïve CD4^+^ T cells can differentiate into different subsets depending on the surrounding cytokine milieu. The different subpopulations show distinct expression patterns of transcription factors and can be characterized by secretion of signature cytokines that are unique for each subset. Each subset takes part in different kinds of immune responses against various pathogens or in mediating autoimmunity.

Nowadays, CD4 T-helper cells are divided into four major subsets, based on their expression profile of transcription factors and secreted cytokines: Th1, Th2, Th17, and regulatory T cells (Treg) (Figure 1). The first two subsets, Th1 and Th2, were identified in the 1980s, when it became clear that CD4^+^ T cells can develop into independent subsets [7]. Th1 cells are characterized by the secretion of IFN*γ*, a proinflammatory cytokine which is necessary for the activation of macrophages and involved in immunity against intracellular pathogens [8, 9]. They have also been linked to cell-mediated autoimmune diseases. Th2 cells produce mainly IL-4, IL-5, and IL-13 and play an important role in allergy as well as in the clearance of various extracellular pathogens and parasites [8, 9].

Figure 1 summarizes key cytokines that drive the differentiation of T-helper cell populations, their main effector cytokines, and the characteristic transcription factors for the different subsets. The differentiation of Th1 cells is mainly induced by IL-12 [9, 10] and can be further enhanced by IFN*γ*. Th2 cells develop in the presence of IL-4 [10]. Th1 and Th2 negatively regulate each other through actions of their specific cytokines: IL-12 represses the induction of Th2 cells, whereas IL-4 inhibits Th1 cell development. On the transcriptional level, Th1 polarizing cytokines induce the transcription factors T-bet and STAT4, whereas Th2 cells require the action of GATA3 and STAT6 [11]. 

Regulatory T cells (Treg) are a unique subset of CD4^+^ T-helper cells that control effector T-cell responses to prevent autoimmune reactions. Activated Treg produce the anti-inflammatory cytokines IL-10 and TGF*β*, thus suppressing the development of functional immune reactions [12]. The differentiation of Treg is induced by TGF*β* [13, 14] but inhibited in the presence of proinflammatory cytokines. Treg cells are characterized by the expression of the transcription factors Foxp3 and STAT5 and the expression of CD25 on their surface [15]. 

Th17 cells are a more recently discovered subset of CD4^+^ T-helper cells characterized by the production of their signature cytokine IL-17. They represent another subtype of proinflammatory T-helper cells that differs from Th1 and Th2 cells in development and function. Differentiation of Th17 cells needs the combined actions of TGF*β*, IL-6, and IL-21 in mice [16–18], whereas IL-6 and IL-21 can be replaced by IL-23 or IL-1*β* in humans. These cytokines induce the expression of the orphan nuclear receptor ROR*γ*t (mice) or RORc (human) [19, 20]. ROR*γ*t (or RORc) is necessary and sufficient for the development of Th17 cells [21], but the transcription factors ROR*α* and STAT3 are also activated [22, 23]. Development of Th17 cells is suppressed by IFN*γ* and IL-4 that promote Th1 or Th2 cells, respectively [24]. TGF*β* alone, in absence of other proinflammatory cytokines like IL-6, induces FoxP3^+^ regulatory T cells instead of Th17 cells, which shows the close relationship between Th17 and Treg. Once Th17 cells have developed, IL-23 is needed for stabilization and further expansion of these cells in mice [25, 26]. For human Th17 cells, also IL-1*β*  and IL-6 can act to enhance development and expansion of these cells. Activated Th17 cells secrete IL-17A, IL-17F, IL-21, IL-22, and TNF*α*, which then promote tissue inflammation by induction of other proinflammatory mediators and recruitment of leukocytes, mainly neutrophils, to the site of inflammation [27, 28]. IL-17 can induce the expression of neutrophil-attracting chemokines, like CXCL1, CXCL2, or CXCL8 in various cell types, among them a variety of epithelial and endothelial cell types, but IL-17 itself can also act to mobilize and activate neutrophils [29, 30].

For nonimmune cells of the liver, there is not much known about the effects of IL-17 so far. It has been shown that stimulation with IL-17 induces the expression of several inflammation-associated genes, including chemokines and C-reactive protein, in primary hepatocytes [31, 32]. These effects can be enhanced by adding TNF*α* or IL-1, and this strongly suggests a role in sustaining liver inflammatory processes for Th17 cells. Human biliary epithelial cells have been described to express IL-6, IL-1*β*, IL-23p19, and several chemokines (CXCL1, CXCL8, CCL20, and others) upon stimulation with IL-17 [33]. As all these cytokines and chemokines are associated with Th17 cells, biliary epithelial cells seem to be able to enhance and sustain Th17-type responses. Interestingly, IL-23p40, the shared subunit of IL-23 and IL-12 that would also favour Th1-type responses, is not upregulated.

Th17 cells have been shown to be involved in the clearance of extracellular bacteria and fungi [34, 35]. They are abundant in the intestinal lamina propria where they are induced by commensal bacteria and function as a barrier against invading pathogens [36], but they have also been linked to several autoimmune diseases. This has first been shown in mice lacking the IL-23 subunit p19 [37], thus not being able to mount effective Th17 responses because IL-23 is needed for stabilization. These animals show normal numbers of Th1 cells but were protected from development of experimental autoimmune encephalomyelitis, the mouse model for multiple sclerosis [37]. Similar observations have been made in IL-17-deficient mice that show less severe autoimmune reactions than wild-type mice [38]. Since their discovery, IL-17-producing T cells have been found to be present at the site of inflammation in several human inflammatory and autoimmune diseases like inflammatory bowel's disease, rheumatoid arthritis, multiple sclerosis, and others [39, 40]. Taken together, these findings suggest that Th17 responses play an important part in inflammatory tissue injury and autoimmunity in humans.

## 3. Th17 Cells in Experimental Murine Liver Injury Models

### 3.1. T-Cell-Mediated Hepatitis

A widely used murine model for T-cell-mediated hepatitis is Concanavalin A- (ConA-) induced hepatitis [41]. Intravenous administration of ConA results in rapid liver inflammation and necrosis, and many features of ConA injury are believed to resemble human autoimmune liver disorders. Establishment of the disease involves a variety of cell types [42] and is dependent on activation of CD4^+^ T cells, as depletion of these cells ameliorates hepatic injury [43]. CD4 T-cell cytokines IFN*γ* and IL-4 have been shown to play a central role in ConA-induced hepatitis [44, 45], whereas the role of IL-17 is less clear, as recent studies have shown controversial effects since IL-17-deficient mice were reported to develop the same level of liver injury as wild-type mice, suggesting that IL-17 plays no role in T-cell-mediated hepatitis [46]; in contrast, two other groups [43, 47] showed that IL-17-deficient mice develop reduced liver injury compared to wild type mice. Although these differences were not as prominent as in IFN*γ*- or IL-4-deficient mice, where induction of liver injury is almost completely suppressed [44, 45], they were still significant. These findings suggest that IFN*γ* and IL-4 are essential for T-cell-mediated hepatitis, whereas IL-17 is less important. This could be due to the fact that IFN*γ* and IL-4 cause many directly damaging effects on the liver, including induction of proinflammatory cytokines and causing hepatocyte apoptosis [45]. IL-17 also activates other cells in the liver to produce proinflammatory cytokines, but it has been reported to have beneficial effects on hepatocyte apoptosis [47, 48]. These antagonizing effects might limit the importance of IL-17 for T-cell-mediated hepatitis.

### 3.2. Parasitic and Bacterial Liver Infections

C57Bl/6 mice infected with the helminth *Schistosoma mansoni *develop mild fibrosing inflammation against parasite eggs in the liver leading to the formation of small granulomas around these eggs [49, 50]. This immunopathology can be markedly aggravated by immunization with soluble egg antigens in complete Freud's adjuvant (CFA) and was originally believed to be Th1 mediated because the disease correlated with high levels of IFN*γ* [51]. Several more recent studies now revealed that the development of these granulomas is primarily dependent on Th17 responses [52–54]. Mice lacking the IL-12p35 subunit, thus able to make IL-23 but not IL-12, are highly susceptible to *Schistosoma* infections with severe immunopathology, whereas mice lacking IL-12p40, therefore incapable of making IL-12 or IL-23, are resistant to this pathology. Consistently, in vivo treatment with neutralizing anti-IL-17 antibodies significantly reduces formation of hepatic granulomas [54]. In IL-23p19 knockout mice, immunopathology after *Schistosoma* infection is not erased but significantly reduced, which goes along with a decrease in IL-17 and IFN*γ* production in granulomas and impaired recruitment of immune cells to the lesions [53]. Taken together, these findings suggest that Th17 responses are essential for the establishment of schistosome egg-induced immunopathology with IL-23 playing a key role. Furthermore, a study in T-bet knockout mice, which are unable to mount Th1 responses, revealed that these mice show more severe immunopathology with higher levels of IL-17 and IL-23p19, indicating that this immunopathology is mediated only by Th17 and that Th1 responses might as well protect against exacerbation of immunopathology by negatively regulating Th17 responses [52].

Generally, *Schistosoma* infection causes only mild immunopathology due to the induction of Th2 cells that fulfil protective functions. Rutitzky et al. used the CFA immunization model eliciting a strong Th1 response, which caused severe immunopathology. However, the recent finding that the aggravation of disease might be rather due to Th17 cells and that Th1 cells are more likely protective in this case suggests that this might not only be true in this experimental setting but also for other natural helminth infections.

IL-17 has also been linked to innate immunity after bacterial infection of the liver, where it seems to fulfil a protective role rather than being responsible for development of immunopathology. It has been reported that not only classical TCR*αβ* T cells are able to produce IL-17 but also some TCR*γδ* T cells that are associated with innate immune reactions [55, 56]. In a model of *Listeria monocytogenes* infection in the liver, IL-17 produced by *γδ* T cells is critical for protective immunity in early stages of the infection [57]. IL-17 expression in the liver increases shortly after infection, and mice lacking IL-17 develop much more severe immunopathology than wild-type mice. The main source for IL-17 in this early stage is *γδ* T cells, and the authors conclude that IL-17 producing *γδ* T cells take part in protective immunity before adaptive Th17 cells appear.

As described above, IL-17 can have opposing effects on different infection models. One possible explanation might be that the time point of IL-17 appearance is different between the two models, which can lead to different functions. In *Listeria* infection, IL-17 is produced by innate immune cells at a very early stage of infection and ameliorates the disease, whereas in infection with *Schistosoma, *mice develop an adaptive immune response where Th17 cells fulfil the functions leading to immunopathology. This might mean that IL-17 can take part in protective processes if released in an early innate environment, but it exerts more aggravating actions in the context of adaptive immune reactions.

### 3.3. Cholestatic and Autoimmune Injury

Primary biliary cirrhosis (PBC) is an autoimmune liver disease in humans characterized by the formation of autoreactive antibodies as well as the damage and loss of small bile duct cells [58, 59]. IL-2R*α* knockout mice have been identified as an animal model for human PBC as they spontaneously produce autoantibodies and develop biliary ductural damage resembling that of PBC patients [60]. This overall increased proinflammatory immune status compared to wild-type mice might very well be due to a loss of functional regulatory T cells. Treg cells need IL-2R*α* (or CD25) to fulfil their anti-inflammatory capacity and prevent autoimmunity. Thus IL-2R*α* KO mice develop spontaneous autoimmune reactions like the ones mentioned above. This provides the opportunity to study underlying mechanisms of immunopathology that would otherwise be obscured by interfering interfering mechanisms exerted by Treg cells.

IL-2R*α* KO mice show altogether elevated levels of Th17 cells, probably due to the missing repressive effect of IL-2 on the induction of Th17 cells accompanied by higher levels of IL-17 in the serum [61]. Furthermore, IL-2R*α* KO mice show a higher frequency of Th17 cells in the liver compared to wild-type mice [62]. Liver Th17 cells from IL-2R*α* KO mice also produce greater amounts of IL-17 than Th17 cells isolated from wild-type mice. The same group also found that in livers of IL-2R*α* KO mice the ratio of Th17 cells to Th1 cells was much higher than in the spleen. Wild-type mice showed the same tendency, although much less prominent, suggesting a preference of Th17 over Th1 responses in the liver. This hypothesis is further supported by the fact that nonparenchymal cells from the liver of wild-type mice can induce splenic CD4^+^ T cells to produce IL-17. However, the mechanisms of Th17 induction in the liver remain unclear. Also, further studies are needed to investigate whether PBC is really caused by Th17 cells and elevation of IL-17, for instance, in IL-2R*α* KO mice that are also unable to produce IL-17.

### 3.4. Toxic Liver Injury

Halothane-induced liver injury is an animal model for drug-induced liver injury. Mice injected intraperitoneally (i.p.) with halothane show elevated levels of serum transaminases and infiltration of immune cells into the liver, which causes mild liver injury [63]. In these mice, plasma levels of IL-17 are increased, and i.p. administration of a neutralizing anti-mouse IL-17 antibody decreases serum AST and ALT levels. Expression levels of proinflammatory cytokines like TNF*α* in the liver were also reduced. Accordingly, administration of recombinant IL-17 elevated plasma transaminases [64], thus aggravating liver injury. This strongly indicates that IL-17 and Th17 cells are involved in halothane-induced liver injury.

## 4. Th17 Cells in Human Liver Diseases

IL-17 producing T helper cells have received a lot of attention since their discovery as a committed lineage of T helper cells in 2005. Since then, they have led to a reevaluation of many disease phenotypes regarding their underlying immunopathophysiology. In human liver disease, there is accumulating evidence for the involvement of Th17 cells in a variety of inflammatory processes in the liver, including all major disease entities such as alcohol induced liver injury, non-alcoholic steatohepatitis (NASH), viral hepatitis, hepatocellular carcinoma (HCC) and primary biliary cirrhosis (PBC) as well as graft rejection after liver transplantation and autoimmune hepatitis. To give a short, coherent overview regarding the potential involvement of Th17 cells, some of the most recent findings of each disease phenotype shall be reviewed in brief individually.

### 4.1. Alcohol-Induced Liver Disease (ALD)

Alcohol induced liver disease is accompanied by a severe secondary inflammatory reaction following the initial phase induced by toxic metabolites derived from the degradation of ethanol by cytochrome CYP2E1 [65]. The severity of inflammation is directly linked to the amount of subsequent liver damage [66]. Degradation of ethanol via cytochrome P450 2E1 furthermore leads to the induction of PPAR*α* signalling as well as formation of reactive oxygen species (ROS) and activation of TNF*α* production and TRAIL-mediated hepatocyte apoptosis [67]. Regarding the infiltration of leukocytes into inflamed liver tissue, T cells have been described as a major part of the inflammatory response following alcohol-induced liver injury, showing high activity for example by secreting a variety of inflammatory cytokines such as IL-1*β*, IL-6, and TNF*α* in vitro after isolation [68]. Furthermore, this infiltration is accompanied by large amounts of neutrophils that also invade the liver tissue and are commonly found in alcohol-induced apoptotic lesions in close association to dying hepatocytes [69, 70]. A recent study by Lemmers et al. could show a close correlation between neutrophil recruitment and the presence of IL-17 producing T-helper cells within the inflammatory liver infiltrates in patients after alcohol-induced liver intoxication [71]. They demonstrated that ALD patients not only showed a significant increase in both IL-17 plasma titers and frequency of IL-17^+^ T cells, but also displayed a correlation between liver infiltration of neutrophils and Th17 cells. Furthermore, they could show that Th17 cells produced IL-8 as well as GRO*α* and that these factors were both necessary and sufficient to induce recruitment of neutrophils [71].

### 4.2. Nonalcoholic Steatohepatitis (NASH)

Obesity accompanied by metabolic syndrome and steatohepatitis is one of the most common causes for chronic liver inflammation leading to progressive tissue injury and liver fibrosis. Albeit the immunopathology of NASH is poorly understood to date, various studies suggest an immediate link between inflammatory response and subsequent liver damage. In general, the induction of misdirected lipid oxygenation by by P450 cytochromes such as CYP2E1 and CYP4A can lead to the formation of free radicals and the formation of reactive oxygen species (ROS), which can act as intrinsic danger signals and triggers of inflammatory responses such as macrophage activation and secretion of cytokines such as TGF*β*, TNF*α*, and IL-6, leading, for example, to activation of TRAIL signaling and hepatocyte apoptosis [72, 73]. Hence, the balance between oxidants and antioxidants critically influences the severity of liver injury, acting as key players in the induction of NASH [73, 74]. The involvement of the adaptive immune response has currently been described in the context of the classical Th1/Th2 paradigm [75], but there is also evidence emerging for a potential involvement of Th17-mediated T-cell responses. For instance, fatty liver-associated inflammation is commonly accompanied by an infiltration of perivenular and periportal infiltration of both neutrophils and lymphocytes, suggesting an enhanced recruitment via their two major receptors CXCR1 or CXCR2 [74, 76]. However, the exact functional role of the neutrophils as well as the recruiting mechanisms still have to be further elucidated, although several studies propose an active involvement rather than being only bystander cells that are recruited due to inflammation [70, 74, 75]. Taken together, the close link between neutrophil infiltration, IL-6 signalling, and Th17 responses [27, 28] and their presence in NASH suggests a functional correlation between fatty liver-induced inflammation and a Th17 immune response.

### 4.3. Viral Hepatitis

In both chronic Hepatitis B (HBV) and Hepatitis C Virus (HCV) infections, recent reports indicate a close correlation between virus-induced liver inflammation, infiltration and activation of Th17 cells and the amount of liver damage caused by the antiviral immune response. For instance, a close correlation between liver infiltrating as well as circulating Th17 cells and the amount of liver damage has been shown in chronically infected Hepatitis B patients. A shift from Th1 to Th17 seems to be potentially disadvantageous for the patient in terms of antiviral defense and liver disease progression, since stronger Th17 responses are associated with higher viral plasma load, increased levels of serum transaminases, and enhanced activation of blood monocytes as well as liver macrophages. Antigen-specific responses of virus-specific Th17 cells have been described for both HBV and HCV, leading to similar pathophysiological changes in both infections [34, 71, 77]. For HBV, HBcAg especially has been shown to be one of the key Th17-inducing antigens, leading to an IL-17R-induced activation of macrophages and monocytes followed by upregulation of CD86, B7H1, B7DC, and CD83 and also cytokines such as IL-1*β*, IL-6, TNF*α*, IL-23p19 and IL-12p35 [78]. Finally, it has been reported that antiviral therapy with pegylated interferon and ribavirin in HCV-infected patients leads to a reduction of both Th1 and Th17 responses, ameliorating HCV-mediated liver inflammation [79].

### 4.4. Hepatocellular Carcinoma (HCC)

In HCC formation, regulating and suppressing antitumoral immune responses is one of the major hallmarks of progressive liver tumor development. Several studies also suggest an involvement of Th17 cells in enhanced tumor formation and survival as well as a negative correlation between tumor-associated Th17 responses and patient survival. As indicated by a study by Hou et al., Th17 cells in general can perform anti-apoptotic functions, which can generally be beneficial for the survival of HCC tumor tissue [80]. Tumor-infiltrating Th17 cells express high levels of CCR4 and CCR6 and therefore respond to tumor-derived CCL20 signals [81]. Furthermore, it has been shown that CD68^+^ HCC-stroma-associated macrophages are able to induce Th17 T-cell responses rather than acting as myeloid-derived suppressor cells (MDSC), therefore potentially directing tumor-associated T-cell responses into a Th17-type direction [82].

The functional role of Th17 cells in supporting tumor formation or survival is only poorly understood up to date and remains somewhat controversial, since in viral hepatitis as well as ALD-Th17 cells have been described to be proapoptotic. It has been suggested that IL-17 and IL-23 can act as angiogenic factors, therefore promoting tumor survival [81, 83–85]. One explanation for these opposed findings could be the fundamentally different disease entity of a liver-damaging agent such as a pathogen or a toxin on the one hand and a relatively immunologically inert or even immunosuppressive environment created by growing tumor tissue on the other hand. On the one hand, danger sensing and inflammation could lead to a feed-forward loop where Th17 cells would aggravate the subsequent inflammation; on the other hand, proangiogenic functions would promote survival of tumor tissue due to enhanced oxygenation, therefore preventing tissue necrosis and activation of immune cells by the release of danger signals.

### 4.5. Primary Biliary Cirrhosis (PBC)

Primary biliary cirrhosis (PBC) is directly linked to a large inflammatory response due to the presence of highly immunogenic danger signals such as malsecreted bile acids, which accumulate in liver parenchyma owing to obstruction of either small or large bile ducts. Several studies have shown a close correlation between formation of PBC and Th17 immune responses in humans, demonstrating a skewed balance from a Treg response towards Th17 in liver infiltrating as well as circulating T-helper cell subtypes [86]. Furthermore, it has also been reported that biliary epithelial cells can directly respond to IL-17, express IL-17R, and upon engagement, start producing acute inflammatory signals such as IL-6, IL-1*β*, and IL-23. Finally, PBC has been described to be accompanied by a direct infiltration of IL-17^+^ cells into damaged bile ducts [33, 62].

### 4.6. Liver Transplantation/Graft Rejection

In liver transplantation and liver graft rejection, only limited evidence exists up to now that suggests an involvement of Th17 cells. However, a study presented by Fabrega et al. describes a possible correlation between acute liver rejection and Th17 induction, showing an overall increase in serum IL-17/IL-23 after transplantation compared to healthy controls in general as well as an even more pronounced IL-17/IL-23 response upon acute graft rejection [87].

### 4.7. Autoimmune Hepatitis

The exact immunopathology of autoimmune hepatitis is still unknown, but the involvement of liver-specific T-cell responses has been suggested for example against liver-derived antigens such as CYP2D6 antigen, a hepatic P450 cytochrome that can act as a class II restricted antigen, leading to the loss of Treg cell function and shifting to an autoimmune T-cell response [88]. Although the exact contribution of Th17 cells to autoimmune hepatitis is unclear, in acute liver inflammation high levels of plasma IL-17 could be detected in patients suffering from autoimmune hepatitis [89].

Taken together, Th17 cells appear to be involved in the pathogenesis of human liver disease, likely mediating distinct immunological functions. On the one hand, Th17 cells are actively engaged in the induction and orchestration of innate immune responses, neutrophil recruitment, and neutrophil activation as commonly seen in ALD, NASH, and PBC. These aberrant inflammatory responses are likely to be critical factors for liver injury and damage progression. On the other hand, Th17 cells can also induce anti-inflammatory responses and drive tissue growth and angiogenesis as seen in HCC. After viral infection with HBV or HCV, it remains to be elucidated whether Th17 cells promote liver damage and are directly involved in enhanced virus survival or if they are simply less capable compared to Th1 T-helper cells in the induction of antiviral responses and therefore are not as competent in limiting disease progression and viral spreading and persistence.

## 5. Interference with Th17 Cell Migration as Potential Therapeutic Target

Lymphocyte migration and chemokine-mediated trafficking has been studied extensively over the last two decades. The rationale for a thorough understanding of lymphocyte migration in various inflammatory conditions is that this would possibly allow interfering with these chemokine pathways as novel therapeutic approaches. Although no chemokine-directed drug has been introduced for the treatment of liver diseases yet, much has been learned about leukocyte trafficking in various experimental settings, leading to the identification of key receptors and their related ligands for migration of lymphocyte subpopulations such as B cells, cytotoxic T cells and T-helper cells in homeostasis and inflammation. For instance, CCR7 and CXCR5 have been described as the most important chemokine receptors mediating lymphocyte traffic towards and within secondary lymphatic organs in constitutive migration and turnover, whereas CCR1, CCR5, CXCR3, CCR3, and CCR4 have clearly been linked to recruitment of T lymphocytes into inflamed peripheral tissues [90].

**Figure 2:**
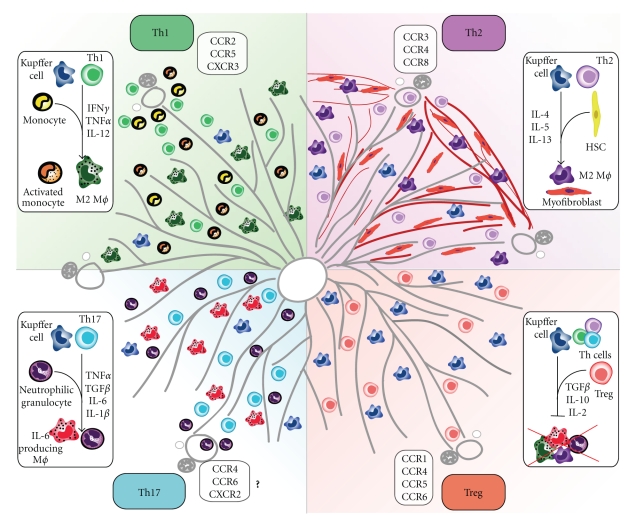
*T-cell-mediated inflammation of the liver. *Four different types of T-helper cell responses have been described to influence various inflammatory processes in the liver. Th1 responses lead to classical activation (M1) of liver-resident macrophages such as Kupffer cells as well as recruitment of monocytes from the bloodstream, promoting a proinflammatory environment by secretion of IFN*γ*, TNF*α*, and IL-12. Th1 infiltration is mediated mainly by engagement of chemokine receptors CXCR3 and CCR5. Th2-type responses are thought to lead to an alternative activation of macrophages (M2) via IL-4 and IL-13, leading to a profibrotic response by activation of hepatic stellate cells (HSC) and inducing their differentiation into myofibroblasts. Th2-type responses are linked mainly to CCR3- and CCR4- mediated chemokine signalling as well as potentially CCR8 under certain conditions. Th17 cell responses in the liver have only recently been described to be involved in various inflammatory processes induced, for example, by alcohol-induced liver disease, HCC, or HBV/HCV-induced hepatitis. Th17 cells lead to activation of macrophages and recruitment of neutrophils, inducing an innate response by secretion of cytokines such as IL-1*β*, IL-6, and TNF*α* but also regulatory factors such as TGF*β*. Recruitment of Th17 cells may be associated with CCR6- and possibly also CCR4-mediated signalling. T regulatory cells (Treg) have been described to be mainly immunosuppressive, secreting anti-inflammatory cytokines such as IL-10 and TGF*β* as well as consuming IL-2, which is a key factor for immunogenic activation of T cells. Therefore, Treg inhibit and suppress T-cell activation and effector functions as well as preventing activation of innate immune cells. A broad variety of chemokine receptors have been linked to Treg migration, for example, CCR1 and CCR4 but also CCR5 as well as CCR6, suggesting a functional overlap for these receptors in different T-helper cell responses.

In particular, the differential migration of T helper cell subsets critically influences the outcome of the subsequent immune response following lymphocyte infiltration. For Th1- as well as Th2-type immune responses, these relationships have been studied in great detail and have been reviewed elsewhere [90–93]. In brief, CCR5 and CXCR3 have been described to be involved in migration of Th1 T-helper cells in various inflammation models, responding to chemokines such as CCL3 (MIP-1*α*), CCL4 (MIP-1*β*), and CCL5 (RANTES) as well as CXCL9 (MIG), CXCL10 (IP-10), and CXCL11 (ITAC) [91, 94–97]. In contrast, CCR3 and CCR4 have been described as the main receptors involved in trafficking of Th2 T-helper cells, responding to signals such as CCL11 (Eotaxin-1), CCL13 (MCP-4), and CCL26 (Eotaxin-3) as well as CCL17 (TARC) and CCL22 (MDC) [91, 95, 98, 99]. Although this has not been fully confirmed for each receptor or ligand in liver disease, experimental evidence supports the assumption that most of these pathways also apply to settings of liver inflammation (Figure 2) [100].

For regulatory T cells the picture is a lot less clear, since there seems to be at least a partial functional overlap between signals that mediate immunity or tolerance. Migration of Treg has been associated with CCR4- as well as CCR5-, CCR6-, and CCR8-mediated signaling and their cognate chemokines, but there seems to be a high degree of variability depending on the type of inflammation and the target tissue in terms of the homing properties of these cells [101–104]. The exact function of Treg in hepatic disease and their migration into the liver is still a matter of debate; however, CCR4 and CXCR3 appear to be involved in Treg recruitment and positioning within the liver [105].

Understanding the migratory fate of Th17 cells has been of particular interest over the last couple of years, since understanding the distribution and recruitment of these cells in homeostasis and inflammation might also shed some light on their immunological function. Because recent reports suggest a coevolution of Treg and Th17 cells [106], it is also suggestive that there is also at least some redundancy in the recruitment of these two T-helper cell subtypes. Indeed, it has become clear that receptors such as CCR6 as well as CCR4 seem to be the main chemokine receptors driving infiltration of Th17 cells in homeostasis and inflammation. In homeostatic conditions, CCR6/CCL20 (MIP3*α*) appears to be the main regulator promoting Th17 cell migration into the Peyer's patches of the gut, where the balance between immigration of Th17 and Treg cells critically influences the development of either immunity or tolerance [107]. The recruitment of Th17 cells into the liver, either in homeostasis or inflammation, has not been studied in great detail yet due to the fact that interest in Th17 cells in liver disease has emerged only over the past few years. 

Various disease models such as experimental autoimmune encephalomyelitis (EAE), systemic lupus erythematosus (SLE), or nephrotoxic glomerulonephritis (NTN) have been linked to infiltration of Th17 cells, most of them reporting CCR6 as a key factor for Th17 infiltration into the target tissues as well as a potential role for CCR4-directed migration [104, 108–112]. Furthermore, priming of Th17 cells has also been described to be critically dependent on CCR7-directed migration of antigen-presenting dendritic cells (DC) and their subsequent production of IL-23p19 in a model of EAE [112]. Functionally, Th17 cells are thought to mainly initiate an innate immune response especially by the recruitment of neutrophils via CXCL8, CXCL2, and CXCL5 directed signalling (targeting CXCR1 and CXCR2), as it has been described for example in models of renal cell carcinoma [113], uveitis [114], or renal inflammation [115]. There is also some evidence for a potential signal amplification loop between neutrophils and Th17 cells, since activated neutrophils also have been reported to produce large amounts of CCL20 under some conditions and therefore potentially also are able to induce or maintain a Th17-type response [116]. In humans, the migratory fate of Th17 cells seems to be less clear up to now, since a variety of receptors associated with either Th1, Th2 or even Treg have been described to be expressed also on Th17 cells such as CCR2, CXCR3, CCR5, CXCR6 but also CCR4 as well as CCR6, CCR7 and CXCR5 [117]. Extensive research activities are currently underway to unravel the migratory pathways of Th17 cells in experimental liver injury and in human liver diseases.

The approach to therapeutically interfere with migratory pathways of Th17 or other immune cell subsets via blocking of chemokine—chemokine receptor interactions is generally appealing, as this could possibly be a highly selective strategy. However, other components of the immune reaction could also serve as therapeutic targets, for example, effector or Th17-differentiation cytokines. For instance, neutralizing antibodies against IL-17 or IL-23 might be useful in liver diseases, while neutralization of IL-6 could be potentially harmful due to its overall protective effects in chronic liver injury [118]. Also, a local redifferentiation or de-novo differentiation of T-helper cell subsets in the inflamed liver seems possible given the differentiation protocols used for in vitro generation of Th17 cells, but would certainly require a better understanding of the appropriate cytokine cocktail and a highly specific delivery system to the injured liver.

## 6. Conclusions and Outlook

Perpetuated inflammation and subsequent hepatic fibrosis are common characteristics of chronic liver diseases in humans and have long been thought to be primarily associated with unbalanced Th1/Th2 responses in the liver. However, this current view may have to be revised in some aspects since the recently discovered Th17 cells may also play an active role in shaping the local inflammatory response in the liver. Their preferred differentiation upon TGF*β* and IL-6 stimulation, two cytokines known to be abundantly present in the injured liver, makes a contribution of Th17 cells to hepatic inflammation very likely. The major hallmark of Th17 cells is the production of cytokines such as IL-17 and IL-22 as well as the recruitment and activation of neutrophils, and they have been described to play important roles not only in host defence against microbial infections but also in tissue inflammation during autoimmunity regarding liver disease; initial studies in humans and mice indeed also revealed activated Th17 cells and Th17-related cytokines in various liver diseases. However, neither the studies linked to human liver diseases such as HBV/HCV infections, NASH, HCC, or toxic liver damage nor functional experiments in different murine models are fully conclusive at present. The exact pathogenic contribution of Th17 cells to liver inflammation might very well vary upon the underlying disease, for example, between infectious and autoimmune disorders. Therefore, it will be of outstanding importance to understand the function of Th17 cells in acute and chronic liver inflammation and also hepatic fibrogenesis as well as the chemokines/chemokine receptors promoting hepatic Th17 cell recruitment in order to gauge whether interference with Th17 migration or differentiation might represent a novel target for the treatment of liver disease.
